# The brain atlas of a subsocial bee reflects that of eusocial Hymenoptera

**DOI:** 10.1111/gbb.70007

**Published:** 2024-11-08

**Authors:** Benjamin C. Pyenson, Jesse L. Huisken, Nandini Gupta, Sandra M. Rehan

**Affiliations:** ^1^ Department of Biology York University Toronto Ontario Canada

**Keywords:** brain evolution, cell type evolution, diapause, neurotransmitters, single cell, sociality

## Abstract

The evolutionary transition from solitary life to group‐living in a society with cooperative brood care, reproductive division of labor and morphological castes is associated with increased cognitive demands for task‐specialization. Associated with these demands, the brains of eusocial Hymenoptera divide transcriptomic signatures associated with foraging and reproduction to different populations of cells and also show diverse astrocyte and Kenyon cell types compared with solitary non‐hymenopteran insects. The neural architecture of subsocial bees, which represent evolutionary antecedent states to eusocial Hymenoptera, could then show how widely this eusocial brain is conserved across aculeate Hymenoptera. Using single‐nucleus transcriptomics, we have created an atlas of neuron and glial cell types from the brain of a subsocial insect, the small carpenter bee (*Ceratina calcarata*). The proportion of *C. calcarata* neurons related to the metabolism of classes of neurotransmitters is similar to that of other insects, whereas astrocyte and Kenyon cell types show highly similar gene expression patterns to those of eusocial Hymenoptera. In the winter, the transcriptomic signature across the brain reflected diapause. When the bee was active in the summer, however, genes upregulated in neurons reflected foraging, while the gene expression signature of glia associated with reproductive functions. Like eusocial Hymenoptera, we conclude that neural components for foraging and reproduction in *C. calcarata* are compartmentalized to different parts of its brain. Cellular examination of the brains of other solitary and subsocial insects can show the extent of neurobiological conservation across levels of social complexity.

## Graphical abstract

Single‐cell gene expression was used to develop the first brain atlas for a small carpenter bee describing neurons and glia cell types for this species. Astrocytes and Kenyon cell types are conserved across Hymenoptera in comparison to Diptera. Gene expression related to foraging and reproduction are separated to different brain cell populations.

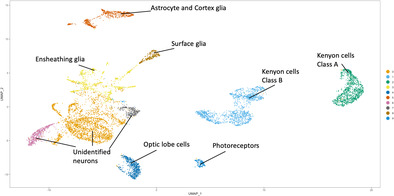

## 1 INTRODUCTION

The brains of many social animals differ in size and structure from those that are not social (^5^; reviewed in^6^). Due to a number of correlated factors, the evolutionary drivers of the social brain can be challenging to identify. Because group size is correlated with brain size in some social vertebrates, the need for communication among group members may have influenced the size of the brain.^7^ Conversely, regions of the insect brain involved in communication and recognition of others depend on group size (reviewed in^8^, ^9^). Associated traits of a social animal's lifestyle can also influence its neural architecture (reviewed in^10^). For instance, the ability to create a nest and the degree of task‐specialization are linked to the size of regions of a social insect's brain.^11^, ^12^ An animal's foraging preferences may also drive brain evolution of social animals: the dietary preferences of mammals is associated with their brain size^13^; and the degree of foraging specialization is linked to the size of brain regions in some beetle and bee species.^14^, ^15^ While the size of specific regions of the brain related to social living is generally dependent on the size of an animal's body,^16^ a brain region can also be influenced by the development of other portions of the brain.^17^, ^18^ These compartments of the brain therefore show allometric relationships that differ by the taxon (reviewed in^8^).

While the sizes of specific regions of the brain differ by an animal's social environment in multiple lineages,^19^, ^20^, ^21^, ^22^ cellular data can suggest cell types that are associated with social behavior.^23^ Gene expression specific to populations of neurons likely emerged from the regulation of genetic programs in ancestral secretory cells.^24^, ^25^ While some vertebrate neuron types are conserved over evolution, the diversification of neuron cell types follows a model of duplication followed by functional divergence of gene regulatory programs.^26^, ^27^, ^28^ In hymenopteran insects, the duplication and divergence model of cell type evolution is also supported for the evolution of Kenyon cells that reside in the Mushroom Body (MB),^29^ a brain region thought to be involved in the recognition of others (reviewed in^30^). Complex animal brains have also evolved a diversity of glia (^31^; reviewed in^32^). One type of glia, astrocytes, have diversified into multiple types in mammals as well as in social insects.^3^, ^33^ The evolution of myelination and ensheathment functions in glia facilitated more efficient information transfer by neurons (reviewed in^34^), suggesting that both neurons and glia could have diversified in response to the cognitive demands of social life.

The brains of solitary animals were thought to differ from those that are eusocial due to life‐history traits,^35^ like a division of reproduction among individuals, overlapping generations and cooperative care of brood.^36^ Diapause, a period of diminished metabolism, behavioral activity and ovarian activity (reviewed in^37^), exerts different effects on solitary and eusocial bees.^38^ For instance, the experience of diapause for larvae of some bee and wasp species determines whether they become queens as opposed to workers.^39^, ^40^

Unlike eusocial organisms, solitary females raise brood without the help of others.^41^ Solitary insects can be distinguished from subsocial ones who extend parental care beyond the brood's egg phase of development (reviewed in^42^). Subsociality is thought to be a prerequisite and evolutionary antecedent to eusocial life from solitary ancestors in Hymenoptera (reviewed in^41^, ^43^). The foraging that contributes to the prolonged brood care behavior of subsociality may have become uncoupled from reproduction in an ancestral insect to facilitate group‐living.^44^ Because brood care is also prominent in eusociality,^36^ the neural architecture of eusocial insects may be shared with a subsocial mother bee that provides extended care for her offspring.

Using the growing number of insect genomes as references for mapping gene transcription,^45^ single‐cell transcriptomics of the insect brain has showed various types of neurons (reviewed in^46^). Visual information is captured by insect photoreceptors that interact with other neurons in the optic lobe, which replicate into adulthood.^47^, ^48^ Optic lobe cells of the pharaoh ant, *Monomorium pharaonsis*, are transcriptionally‐similar to the fruit fly, *Drosophila melanogaster*.^4^ Neurophysiological activity in these cells precedes attention to visual stimuli in both the honey bee, *Apis mellifera* and *D. melanogaster*, suggesting highly conserved function.^49^, ^50^ In *D. melanogaster*, three types of Kenyon cells (e.g. α/β, α′/β′, γ^51^), are involved in learning and memory.^52^ In contrast, four types of Kenyon cells have been identified in *A. mellifera*, consisting of class II neurons and three categories of class I neurons (large, medium, small^53^).

The relative abundance of neurons that synthesize and release specific neurotransmitters can be associated with specific neurophysiological and behavioral functions in insects.^54^, ^55^ Acetylcholine is excitatory, stimulating body growth and food intake in *D. melanogaster*.^56^ Gamma‐aminobutyric acid (GABA) is an inhibitory neurotransmitter related to learning in *D. melanogaster*,^57^ but also feeding in *D. melanogaster* and in *A. mellifera*.^58^, ^59^ In these species, glutamate, another neurotransmitter, can be inhibitory in the antennal lobe.^60^, ^61^ Monoaminergic neurons, comprising those that release the neurotransmitters serotonin, dopamine, tyramine, octopamine, and histamine, are related to aggression in many insects (reviewed in^62^), but seem to only be linked to reproduction in social ones (reviewed in^63^). In *D. melanogaster* and *A. mellifera*, the abundance of classes of neurons that express neurotransmitters are similar: cholinergic neurons are the most abundant and monoaminergic neurons are the rarest; and GABAergic and glutamatergic neurons show intermediate abundance.^55^, ^64^

Non‐neuronal cells in the insect brain vary in their location and function. Glia relate to homeostasis and consist of cortex glia, ensheathing glia, astrocytes, and surface glia in solitary and social insects (^4^; reviewed in^32^). Cortex glia cover somata of neurons, astrocytes contact synapses, and ensheathing glia ensconce axons of *D. melanogaster* (reviewed in^32^). Surface glia, comprised of perineurial and subperineurial subtypes, comprise the blood–brain barrier that regulates the traffic of molecules like hemocyte cells of the immune system between the brain and hemolymph.^65^ In addition to glia, hemocytes have been found in the brain of *D. melanogaster* as well as *A*. *mellifera*.^1^, ^48^

The expression of genes characteristic of specific insect brain cell types have been used to demarcate homologous cell types in the brains of solitary and social insect species.^4^, ^48^, ^66^ For instance, multiple astrocyte cell types exist in eusocial insects like the Indian jumping ant *Harpegnathos saltator*, *M. pharaonsis* and *A. mellifera* compared with the solitary *D. melanogaster*.^1^, ^3^, ^4^, ^48^ Among neurons, Kenyon cells can be divided into two broad classes, A and B.^4^ Kenyon cells Class B (KCB) consist of class I‐small, class I‐medium and class II Kenyon cells of *A. mellifera* as well as α′/β′ and γ types of *D. melanogaster*.^4^ The diverse class I Kenyon cells of eusocial Hymenoptera likely originated from an increase in the number of cell types linked to parasitism in Apocrita, followed by at least a second diversification event associated with nidification in Aculeata.^67^

In contrast to the expression of reproductive genes that is similar across brain cell types of *D. melanogaster*,^2^ genes linked to foraging and reproduction are expressed in distinct population of brain cells of eusocial insects that resemble brain compartments.^1^, ^64^ As an example, the reproductive activity of individual *H. saltator* and *A. mellifera* is associated with specific transcriptomic signatures in ensheathing glia.^3^, ^64^ Foraging and non‐foraging workers of the Florida carpenter ant, *Camponotus floridanus*, however, differ in the gene expression of their surface glia.^68^ It remains unclear if this transcriptomic compartmentalization of reproduction and foraging also exists in the brain cells of non‐eusocial Hymenoptera.

Because the brain of a subsocial insect represents a simple form of insect sociality,^41^ the subsocial hymenopteran brain may share characteristics with social Hymenoptera. The small carpenter bee, *Ceratina calcarata*, shows subsociality by caring for its offspring as they develop, especially by provisioning them with food as adults.^69^ Brain gene expression, neuroanatomy and biogenic amine examination of *C. calcarata* suggest that learning is associated with foraging behavior.^70^, ^71^, ^72^, ^73^ Other work has characterized the gene expression in the brain during a quiescent diapause state while overwintering.^74^ The reference genome models assembled for this species allow mapping of transcriptomes from single‐cell analysis.^75^, ^76^

Using bioinformatic techniques, we evaluate if the diversity of cell types observed in the eusocial hymenopteran brain is present in a less socially complex insect with rudimentary behaviors needed for group‐living. We first characterize the cell types in the *C*. *calcarata* brain by integrating samples from adult females collected during the summer, when they are reproductive and foraging, as well as those from winter when the bee undergoes diapause. Then, we analyze gene expression and enrichment across cell types to evaluate where the transcriptomic signals for reproduction, foraging and diapause emerge in the brain. Finally, we assess the transcriptional similarity of groups of brain cells in *C. calcarata* to cell types in solitary *D. melanogaster* and in the eusocial aculeate Hymenoptera species *A. mellifera*, *H. saltator* and *M. pharaonsis*.

## 2 MATERIALS AND METHODS

Detailed methods on field collection, data processing and bioinformatics are provided in the supplement. We collected samples of six *C*. *calcarata* adult females from raspberry and sumac stems in Toronto, Canada at two time points in 2022 (Table S1). The two time points comprised a winter sample of three bees from one nest and three bees from three additional nests when they underwent diapause (Biosample: SAMN38226699); and a summer sample of six reproductive and foraging mothers each from different nests (Biosample: SAMN38227370). From fresh‐frozen brains of each sample, personnel from the Princess Margaret Genomics Centre isolated, barcoded and lysed nuclei with 10X Genomics technology to prepare dual index paired‐end cDNA libraries for sequencing. We then mapped reads to the *C. calcarata* genome,^75^ and filtered the gene expression matrices of ambient RNA, empty droplets and doublets.^77^ We then normalized counts for variation in the sequencing depth of nuclei by fitting counts to a negative binomial model according to recommended techniques with the package SCTransform^78^, ^79^, ^80^ in Seurat (v. 4.3.0;^81^) in R (v. 4.2.3,^82^).

With canonical correlation analysis in Seurat,^83^ we then created an integrated object of 8495 nuclei and 13,374 genes to account for differences in each sample. Using a gene annotation of *C. calcarata* supplemented by 8 *D. melanogaster* and 82 *A. mellifera* orthologs, we identified markers of hemocyte, glia and neuron cell types in the *C. calcarata* object.^63^, ^64^ Using highly‐variable genes to calculate principal components, we then clustered the nuclei of the *C. calcarata* object in two dimensions of variance with the Louvain algorithm using Seurat::FindClusters. From violin plots, we identified and removed one cluster of 145 hemocytes before re‐clustering the remaining 8350 nuclei into 10 clusters of neurons and glia cell types. We compared the relative gene expression of orthologs in each cluster to all other nuclei to label them as cell types, a recommended method that balances precision and recall.^84^ To characterize gene functions in each *C. calcarata* neuron and glia cell type associated with diapause in the winter as well as reproduction and foraging in the summer, we calculated Gene Ontology (GO) enrichment in each cell type with topGO (v. 2.48.0,^85^) between the summer and winter samples from the associated GO annotations of differentially‐expressed genes (DEGs) calculated using Wilcoxon tests in Seurat.^86^

Using orthologs of *D. melanogaster*, we then estimated the proportion of neurons involved in the metabolism of different classes of neurotransmitters in the brains of different species. For *C. calcarata* (this study), *H. saltator*,^3^ and *A. mellifera*,^1^, ^29^, ^86^ we estimated orthologs related to the synthesis and release of neurotransmitters with BLAST (v. 2.14.0,^87^; Table S22). We used *D. melanogaster* orthologs for *M. pharaonsis* calculated in another study.^4^ Using Seurat, we then estimated the abundance of cholinergic, GABAergic, glutamatergic and monoaminergic neurons relative to all neurons in the brain for cell‐type atlases of *C. calcarata*, *A. mellifera*,^1^ *M. pharaonsis*,^4^ *H. saltator*,^3^ and *D. melanogaster*.^2^

**FIGURE 1:**
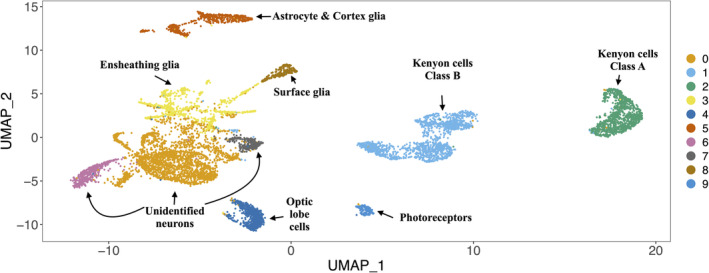
The brain atlas of 8350 nuclei, divided into 10 clusters using Seurat that are then annotated with cell types using the relative expression of canonical markers, some of which are mentioned in Table 1. The cluster number is listed in the legend and the corresponding cell type of each cluster is indicated.

To evaluate the degree of analogy of the cell types in the *C. calcarata* brain to those in the brains of solitary and social insects, we then re‐clustered specific cell type clusters from the integrated brain atlas (Figure 1) into subclusters and assessed the similarity of the transcriptional signatures of *D. melanogaster* orthologs to brain cell types from other species. Specifically, we used Metaneighbor (v. 1.16.0,^88^) to calculate AUROC scores that indicate the transcriptional similarity of *C. calcarata* subclusters to clusters of solitary *D. melanogaster* ^2^ and social Hymenoptera that include *M. pharaonsis*,^4^ *H. saltator*,^3^ and *A. mellifera*.^1^, ^29^, ^86^

## 3 RESULTS

### 3.1 Integrated *C. calcarata* brain atlas and comparison to other insect brains

**FIGURE 2:**
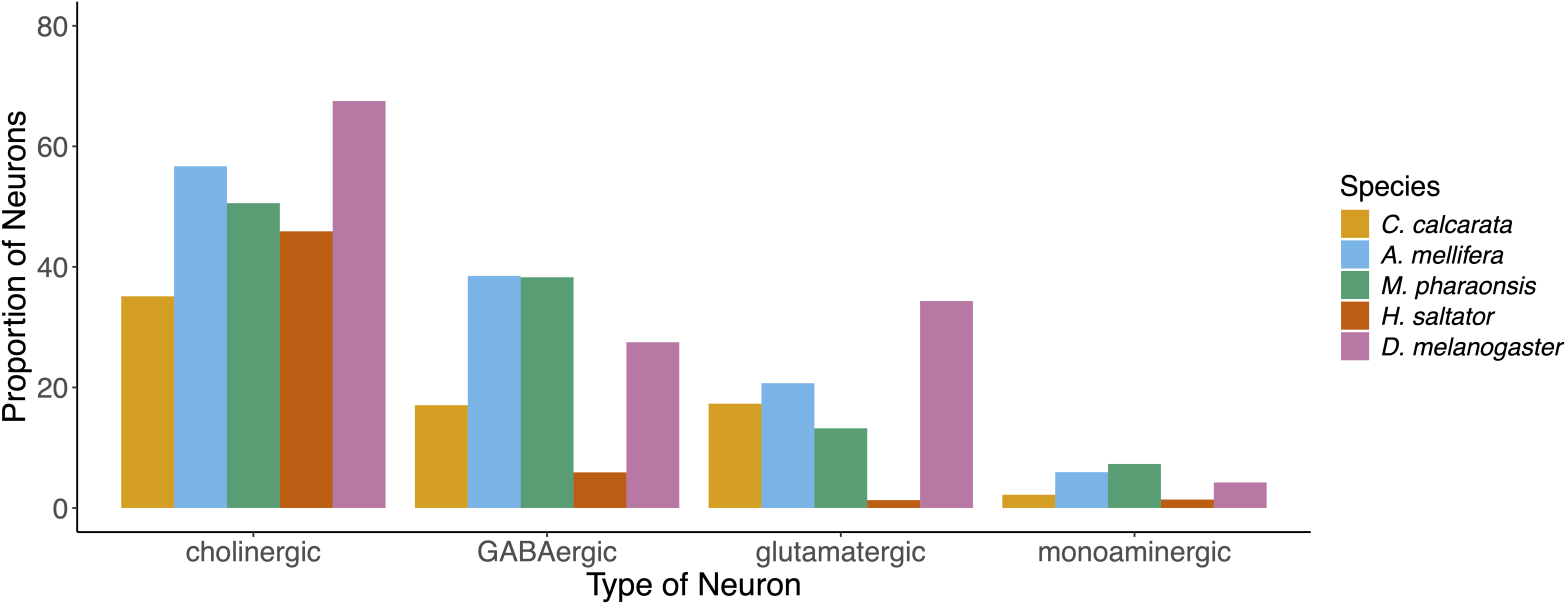
Proportion of neurons that express genes indicating the synthesis and/or release of classes of neurotransmitters estimated from the brain atlases of *C. calcarata* (this study), *A. mellifera*,^1^ *M. pharaonsis*,^4^ *H. saltator*,^3^ and *D. melanogaster*.^2^

We integrated the gene expression of 13,374 annotated genes across 8495 nuclei from the brains of bees collected in the winter and in the summer to create an atlas of neuron and glia cell types of *C. calcarata*. After quality‐control filtering, we clustered 8350 nuclei into seven neural and three glial cell types based on the expression of orthologs that serve as markers for specific cell types (Figure 1). Kenyon cells, photoreceptors and non‐photoreceptor optic lobe cells could be identified among the neurons, while glia consisted of astrocytes, cortex, surface and ensheathing glia (Table 1). The proportions of neurons that express markers indicative of the synthesis and/or release of different classes of neurotransmitters is similar in the *C. calcarata* brain and those of *D. melanogaster*, *H. saltator*, *M. pharaonsis* and *A. mellifera*: cholinergic neurons are the most common; GABAergic and glutamatergic neurons are less abundant; and monoaminergic neurons are the rarest (Figure 2).

**TABLE 1:** The cluster number, cell‐type label and notable markers for each cluster of 8350 total cells in the integrated *C. calcarata* brain atlas.

Cluster number	Cell type	Notable markers	Winter cells	Summer cells	DEGs upregulated in winter	DEGs upregulated in summer
0,6,7	Unidentified neurons	*nSyb*, *Syt1*, *elav*	1482	1809	116	90
1	Kenyon cells‐Class B (KCB)	*mub*, *tk*	721	808	53	49
2	Kenyon cells‐Class A (KCA)	*CamKii*, *Mblk‐1*, *mub*, *tk*, *Fas2*	530	548	**150**	**182**
3	Ensheathing glia	*idgf4*; *tsf1*	442	462	**50**	**71**
4	Optic lobe cells (non‐photoreceptors)	*Nlg2*; *SoxN*; *Lim1*	361	245	107	88
5	Cortex glia/astrocytes	Cortex: *wrapper*, *zyd* Astrocyte: *Eaat1*, *Gs2*	372	180	116	108
8	Surface glia	*Tret1*, *svp*; *Mdr49*	85	152	286	157
9	Photoreceptors	*ninaB*, *ninaC*, *trp*	44	109	162	65

*Note*: The quantity of cells in each cluster as well as the differentially expressed genes (DEGs) significantly upregulated in the winter sample compared with the summer sample are also indicated for each cluster.

*Note*: Bold indicate a group of cells where more genes are upregulated in the summer rather than in the winter sample.

### 3.2 Comparison of gene expression in brain cell types between the summer and winter samples

**FIGURE 3:**
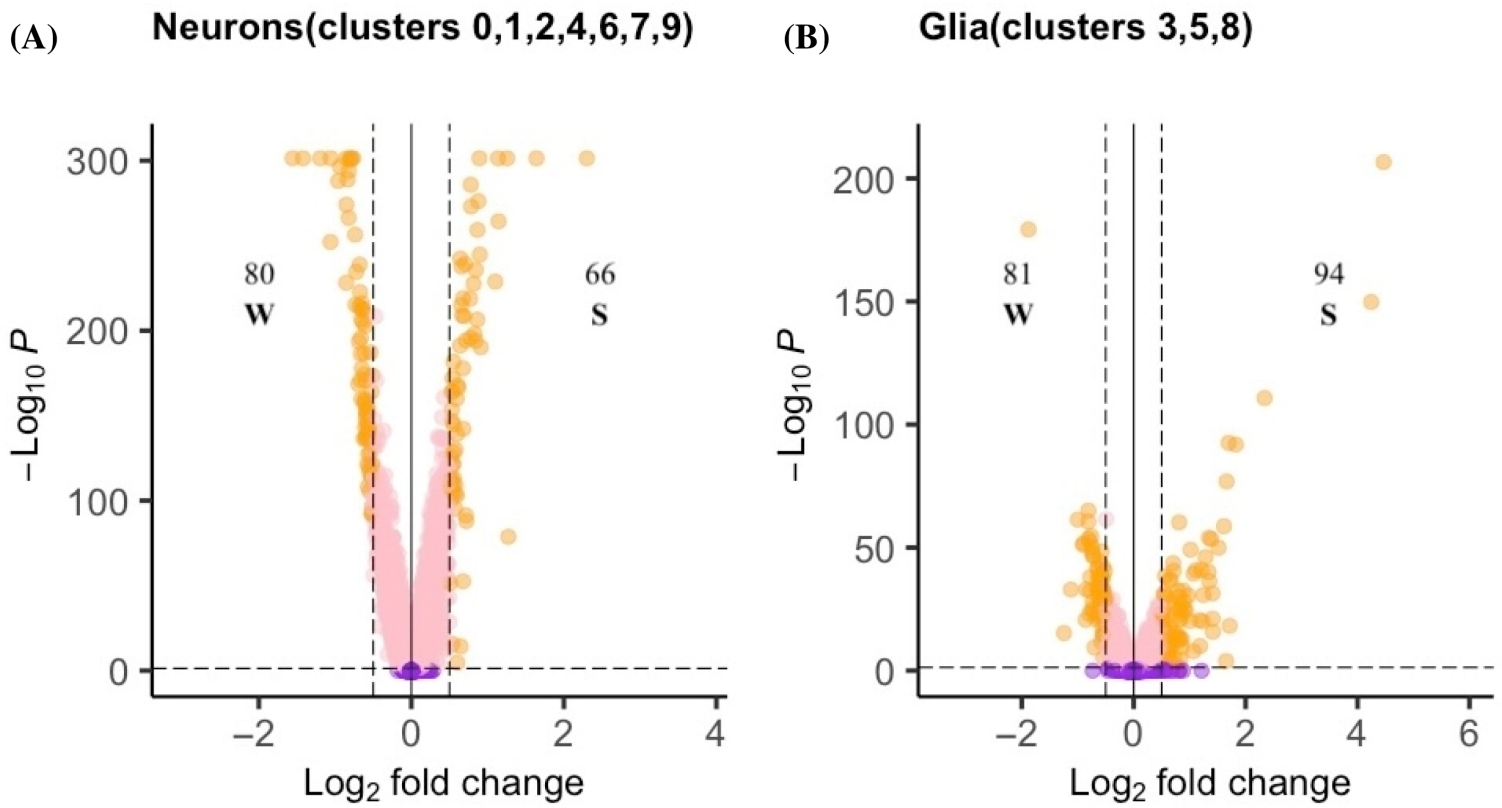
Volcano plots showing log_2_ fold change difference in normalized expression on the x‐axis of 13,374 genes between the winter (W) and summer (S) samples. Significance indicated on the y‐axis by −log_10_ adjusted *p*‐values using Bonferroni correction (i.e. false detection rate, FDR) for genes in the integrated brain atlas of (A) 6657 neuron cells combined from clusters 0, 1, 2, 4, 6, 7 and 9 in Figure 1; and (B) 1693 glia cells combined from clusters 3, 5 and 8 in Figure 1. Negative log_2_ fold change indicates upregulation in W, whereas positive values indicate upregulation in S. Orange indicates those genes judged to be significantly differentially expressed and significantly upregulated to winter or summer with FDR <0.05 demarcated with the horizontal dashed line and log_2_ fold change >0.05 or < −0.05 indicated by the vertical dashed line. Purple indicates those genes with a FDR ≥0.05, while pink indicates those genes with FDR <0.05, but log_2_ fold change ≥ −0.5 and ≤0.5. The number of significantly differentially‐expressed genes that are upregulated in each phenotype is indicated.

To evaluate how gene expression changes in *C. calcarata* brain cell types as it progresses from an overwintering diapause phenotype to a reproductive and foraging one, we calculated the number of differentially expressed genes (DEGs) and the enrichment of GO terms linked to these genes between the summer and winter samples in the integrated brain atlas. Sixty‐six DEGs were significantly upregulated in the summer sample in all neurons, including *apisimin* and the potassium voltage‐gated channel protein Shab (*shab*) (Figure 3, Table S23). Neurons showed GO term enrichment for TORC1 signaling (GO:0038202) and various metabolic or biosynthetic processes (Tables 2 and S25). A total of 182 DEGs were upregulated in the summer sample in Kenyon cells‐Class A (KCA) that were enriched for visual learning (GO:0008542; Tables 2 and S25). This enrichment is supported by the expression of fasciculation and elongation protein zeta‐2 (*fez2*) and the calcium‐activated potassium channel slowpoke (*slo*) (Tables 2 and S23). In non‐photoreceptor optic lobe cells, 88 DEGs were upregulated in the summer sample. Cognition (GO:0050890) was enriched in these cells, which is supported by the upregulation of scoloptoxin SSD14 and the sodium channel protein 60E (*NaCP60E*; Table 2). Tachykinin receptor signaling (GO:0007217) and neuropeptide signaling (GO:0007218) were also enriched in non‐photoreceptor optic lobe cells, which is supported by two DEGs: the SH2 domain‐containing adapter protein F (*shf*); and the programmed cell death protein 2‐like (*pdcd2l*). Optic lobe cell enrichment also included positive regulation of axon guidance (GO:1902669) and positive regulation of canonical Wnt signaling pathway (GO:0090263; Tables 2 and S25). Sixty‐five DEGs were upregulated in the summer sample in photoreceptor cells. Photoreceptor enrichment included dendrite development (GO:0016322), regulation of multicellular organism growth (GO:0040014), neuron remodeling (GO:0016322), steroid hormone mediated signaling pathway (GO:0043401), torso signaling pathway (GO:0008293) and positive regulation of ERK1 and ERK2 cascade (GO:0070374; Table 2). Enrichment for neuron remodeling was supported by *slo*, while the enrichment for steroid hormone mediated signaling pathway was underpinned by expression of retinal degeneration B (*rdgB*), peroxisomal acyl‐coenzyme A oxidase 3 (*acox3*) and catenin delta‐2 (*ctnnd2*). Enrichment for torso signaling pathway and positive regulation of ERK1 and ERK2 cascade was supported by the expression of one gene of unknown function and inositol hexakisphosphate and diphosphoinositol‐pentakisphosphate kinase (*i(1)G0196I*; Table 2).

**TABLE 2:** Gene Ontology (GO) term enrichment in the summer sample for different brain cell types in *C. calcarata*. Cell cluster number refers to Table 1.

GO	Term	Cell type	Cell cluster #
GO:1901136	Carbohydrate derivative catabolic process	Neurons	0,6,7,1,2,4,9
GO:0009262	Deoxyribonucleotide metabolic process		
GO:0008299	Isoprenoid biosynthetic process		
GO:0038202	TORC1 signaling		
GO:0006520	Cellular amino acid metabolic process	Neurons Kenyon cells	0,6,7,1,2,4,9 1,2
GO:0006189	de novo' IMP biosynthetic process		
GO:0005977	Glycogen metabolic process		
GO:0045725	Positive regulation of glycogen biosynthetic process		
GO:0006268	DNA unwinding involved in DNA replication	Kenyon cells	1,2
GO:0000161	Osmosensory signaling MAPK cascade		
GO:0008542	Visual learning	Kenyon cells‐Class A (KCA)	2
GO:0090263	Positive regulation of canonical Wnt signaling pathway	Kenyon cells Optic lobe cells	1,2 4
GO:0050890	Cognition	Optic lobe cells	4
GO:0007218	Neuropeptide signaling pathway		
GO:1902669	Positive regulation of axon guidance		
GO:0007217	Tachykinin receptor signaling pathway		
GO:0016358	Dendrite development	Photoreceptors	9
GO:0016322	Neuron remodeling		
GO:0070374	Positive regulation of ERK1 and ERK2 cascade		
GO:0040014	Regulation of multicellular organism growth		
GO:0043401	Steroid hormone mediated signaling pathway		
GO:0008293	Torso signaling pathway		
GO:0007618	Mating	Glia Astrocyte/Cortex	3,5,8 5
GO:2000130	Positive regulation of octopamine signaling pathway		
GO:0060279	Positive regulation of ovulation		
GO:0046692	Sperm competition		

In glia, 94 genes including ecdysteroid UDP‐glucosyltransferase (*egt*) and vitellogenin (*Vg*) were significantly upregulated in the summer sample (Figure 3). Glia enrichment included mating (GO:0007618), sperm competition (GO:0046692), as well as the positive regulation of ovulation (GO:0060279) and the positive regulation of the octopamine signaling pathway (GO:2000130; Table S25). This enrichment was underpinned by the expression of three genes of unknown function as well as major royal jelly protein 1 (*mrjp1*; Table 2). The cluster of astrocyte and cortex glia cells showed 108 DEGs upregulated in the summer sample. These cells were also enriched for the regulation of octopamine signaling and ovulation (GO:0060279; GO:2000130; Table S25). This enrichment was supported by one gene of unknown function, *mrjp1*, as well as two other genes: collagen alpha chain (CG42342); and the immunoglobulin domain‐containing protein (*oig‐4*; Table 2).

In neurons, 80 DEGs were upregulated in the winter sample. These were enriched for cellular nitrogen compound biosynthetic process (GO:0044271) and various metabolic processes (Table S26). Enrichment in both clusters of Kenyon cells included positive regulation of phagocytosis (GO:0050766) and signaling (GO:0023052). In KCA, 150 DEGs were upregulated in the winter sample, including ubiquitin carboxyl‐terminal hydrolase 34 (*usp34*; Table S24). *Usp34* is also included in the 162 DEGs upregulated in photoreceptors in the winter sample that were enriched for gene expression (GO:0010467), cytolysis (GO:0019835), negative regulation of neural apoptotic process (GO:0043524) and negative regulation of multicellular organism growth (GO:0040015; Table 3).

**TABLE 3:** Gene Ontology (GO) term enrichment in the winter sample for different brain cell types in *C. calcarata*. Cell cluster number refers to Table 1.

GO	Term	Cell type	Cell cluster #
GO:0044271	Cellular nitrogen compound biosynthetic process	Neurons	0,6,7,1,2,4,9
GO:0006071	Glycerol metabolic process		
GO:0006796	Phosphate‐containing compound metabolic process		
GO:0019805	Quinolinate biosynthetic process	Neurons Kenyon Cells	0,6,7,1,2,4,9 1,2
GO:0006114	Glycerol biosynthetic process	Kenyon Cells	1,2
GO:0046168	Glycerol‐3‐phosphate catabolic process		
GO:0006072	Glycerol‐3‐phosphate metabolic process		
GO:0050766	Positive regulation of phagocytosis		
GO:0023052	Signaling		
GO:0019835	Cytolysis	Photoreceptors	9
GO:0010467	Gene expression		
GO:0040015	Negative regulation of multicellular organism growth		
GO:0043524	Negative regulation of neuron apoptotic process		
GO:0006950	Response to stress	Glia Surface Glia	3,5,8 8
GO:0032922	Circadian regulation of gene expression	Surface Glia	8
GO:0043153	Entrainment of circadian clock by photoperiod		
GO:0008286	Insulin receptor signaling pathway		
GO:0048519	Negative regulation of biological process		
GO:0016055	Wnt signaling pathway		
GO:0051782	Negative regulation of cell division	Glia Ensheathing Glia	3,5,8 3
GO:0040017	Positive regulation of locomotion	Ensheathing Glia	3

Eighty‐three DEGs were upregulated in glia in the winter sample including 97 kDa heat shock protein (*hsp110*) and held out wings (*how*). Glia were enriched for response to stress (GO:0006950) as well as negative regulation of cell division (GO:0051782). The latter enrichment was supported by 40S ribosomal protein S5 (*rps5*) and hydroperoxide glutathione peroxidase (*gpx4*; Table 3). Fifty DEGs were upregulated in the winter sample in ensheathing glia (Table 1). Ensheathing glia enrichment for positive regulation of locomotion (GO:0040017) as well as negative regulation of cell division (GO:0051782) was supported by 60S ribosomal protein L9 (*rpl9*) and *gpx4* (Table S24). Of the 286 DEGs upregulated in surface glia cells in the winter sample, enrichment includes the response to stress (GO:0006950), negative regulation of cell division (GO:0051782), insulin receptor signaling pathway (GO:0008286), circadian regulation of gene expression (GO:0032922) and entrainment of circadian clock by photoperiod (GO:0043153; Table S26). Insulin receptor signaling pathway (GO:0008286) enrichment of surface glia cells was supported by five genes: RCC1 and BTB domain‐containing protein 1 (*rcbtb1*); trifunctional enzyme subunit alpha 2C mitochondrial (*hadha*); U2 snRNP‐associated SURP motif‐containing protein (*u2SURP*); thioredoxin reductase 1%2C mitochondrial (*trxr‐1*); and probable G‐protein coupled receptor 158 (*gpr158*). Enrichment of circadian regulation of gene expression (GO:0032922) and entrainment of circadian clock by photoperiod (GO:0043153) were supported by a gene of unknown function, *Hadha* and ceramide glucosyltransferase (*ugcg*; Tables S24 and S26).

### 3.3 Transcriptional similarity to solitary and eusocial species

**FIGURE 4:**
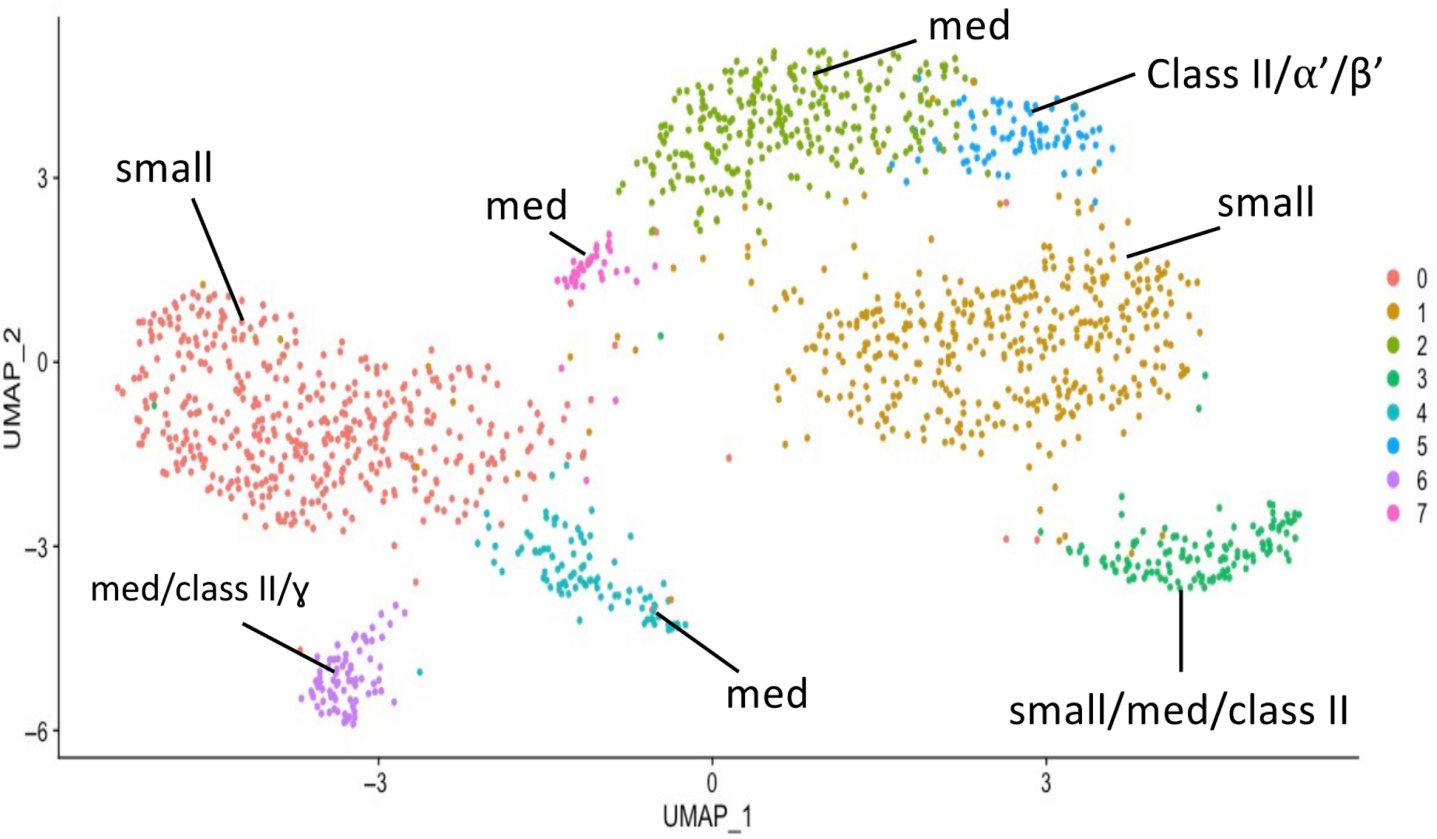
Putative identities of subclusters of *C. calcarata* KCB (Figure 1—cluster 1) inferred from the transcriptional similarity calculated with MetaNeighbor of 4718 orthologs to small, medium and class II Kenyon cell types of *A. mellifera* ^29^, ^64^, ^86^ and from the similarity of *C. calcarata* to α′/β′ and γ Kenyon cell types from *D. melanogaster*.^2^ Number in the legend indicates the number of the subcluster referred to in the text. Small = small Kenyon cells; med = medium Kenyon cells.

To evaluate the degree to which *C. calcarata* cell types are analogous to brain cell types of other insects, we subclustered the cell types identified in the integrated *C. calcarata* atlas and compared the expression of genes in these subclusters to orthologs in the cell types of *A. mellifera, H. saltator, M. pharaonsis* and *D. melanogaster*. Distinct *C. calcarata* subclusters of the KCB could be annotated to KCB cell types from two *A. mellifera* ^29^, ^86^ and one *D. melanogaster* ^2^ datasets. All eight *C. calcarata* subclusters were highly similar to one or more KCB types of *A. mellifera*: small; medium; and class II (Figure 4, Table S21). Subclusters 5 and 6 also showed high transcriptional similarity to the *D. melanogaster* KCB types α′‐ß′ and γ, respectively.

To identify specific neuron cell types not apparent in the *C. calcarata* integrated atlas, we compared subclusters of the unidentified neurons (clusters 0, 6 and 7) to olfactory projection neurons, monoaminergic and unidentified neuron cell clusters identified in datasets from other species (Tables S20, S21). The subclusters 6 and 12 from *C. calcarata* could be unambiguously annotated to the anterodorsal and lateral olfactory projection neurons, respectively, from *A. mellifera* (Figure S1A, Table S21). Olfactory projection neuron clusters of *A. mellifera* were also similar to *C. calcarata* subclusters 9, 11 and 16 at lower AUROC values. These *C. calcarata* subclusters, however, also showed similarity to other types of neurons and were therefore not annotated as olfactory projection neurons. Analogously, clusters of olfactory projection neurons from *M. pharaonsis*, *H. saltator* and *D. melanogaster* were similar to *C. calcarata* subclusters 4, 10, 13 and 14, but showed AUROC values less than 0.9. These subclusters also showed similarity to different neuron cell types, making their characterization as olfactory projection neurons less clear. In contrast, *C. calcarata* subclusters 8, 13, 14, 16 and 17 were annotated as histaminergic because of their high similarity with at least one histaminergic neuron cluster from *A. mellifera* (Figure S1A).

We then divided the combined cluster of astrocytes and cortex glia of the integrated *C. calcarata* brain atlas (cluster 5) into subclusters to determine whether these nuclei were similar to astrocyte and cortex glia cell types from other species (Figure S1B, Table S21). Cortex glia from other species showed high similarity to subcluster 5 of *C. calcarata*. Additionally, subcluster 5 showed upregulation of the canonical markers of cortex glia, *wrapper* and *zyd* (Table S28). Subclusters 1 and 3 showed similarity to cortex glia of other species, but also to astrocyte glia, making the cell‐type annotation of these subclusters difficult. Subclusters 0, 2 and 6 were only similar to astrocyte glia clusters from other species (Table S21). Each of these subclusters showed similarity to different combinations of astrocyte glia cell types of *H. saltator*, *M. pharaonsis* and *A. mellifera*. Only subclusters 0 and 2 showed similarity to the same astrocyte glia cell type of *D. melanogaster*. (Figure S1B). Therefore, three *C. calcarata* subclusters show transcriptional similarity to the astrocytes of eusocial insects, whereas only two subclusters show similarity to solitary insect astrocytes.

Because multiple types of surface glia have been characterized in other insects, we divided the surface glia cluster of the integrated brain atlas of *C. calcarata* (cluster 8) into subclusters and compared these to perineurial, subperineurial and unknown glia cell types of other species (Figure S1C, Table S21). Subcluster 2 of *C. calcarata* is similar to perineurial clusters from *A. mellifera* as well as *D. melanogaster*, while subcluster 1 is similar to a subperineurial cluster from *A. mellifera* as well as a general surface glia cluster from *M. pharaonsis*. Although subcluster 3 shows similarity to two different *D. melanogaster* subperineurial clusters, it also shows high similarity to unknown glia from ants, making its characterization in *C. calcarata* challenging.

## 4 DISCUSSION

Our results suggest that the transcriptomic compartmentalization to different cell types observed in eusocial insect brains are shared in a subsocial bee. After integrating single‐cell brain transcriptomes from summer and winter samples of *C. calcarata*, we created a cellular brain atlas of a subsocial hymenopteran insect consisting of neurons, glia and hemocytes. We found that the proportion of neurons linked to specific neurotransmitters was similar to that of other insects. While we found transcriptional similarity of both neuron and glial cell clusters in the *C. calcarata* brain to cell types from other insect brains, *C. calcarata* cell clusters could be most clearly annotated to the Kenyon cells, astrocytes and surface glia cell types of eusocial insects. Transcriptomic signals of the response to cold temperature were found in neurons and glia in the winter sample. In contrast, the gene expression in the summer sample suggests that reproduction functions may be localized to glia cells, whereas foraging may be attributable largely to neurons.

### 4.1 Conserved cell types among insects

With analysis of single‐nucleus transcriptomic data, we created a cell‐type atlas for the brain of female *C. calcarata* that encompasses gene expression during adult diapause in the winter as well as during the development of reproduction, foraging, mating and flight in the summer.^69^ From the expression of canonical markers in the brains found in other insects, we characterized *C. calcarata* clusters as Kenyon cells, photoreceptors, non‐photoreceptor optic lobe neurons, hemocytes and glia that include ensheathing, cortex, astrocytes and the subperineurial and perineurial groups of surface glia (Figures 1 and S1). Hemocytes have been identified in the brains of *A. mellifera* and *D. melanogaster*,^2^, ^64^ but have not yet been reliably identified in ants.^3^, ^4^, ^68^ Optic lobe cell types show high transcriptional similarity across *A. mellifera* and *D. melanogaster*, supporting their high conservation among insects.^4^ Using estimates of transcriptional similarity to *A. mellifera* and *D. melanogaster*, *C. calcarata* neurons from the antennal lobe that project medially could also be distinguished from those that project anterodorsally (Figure S1A, ^1^, ^48^, ^89^). Histaminergic *C. calcarata* neurons were also identified based on their transcriptional similarity to those of *A. mellifera*.

The proportion of neurons involved in the metabolism of different classes of neurotransmitters was similar between *C. calcarata* and other insects (Figure 2). Cholinergic neurons are involved in the regulation of nutrient‐dependent pathways in *D. melanogaster*,^56^ while GABAergic neurons relate to feeding across insects.^58^, ^59^ The monoaminergic neurons in *C. calcarata* may regulate behaviors such as aggression, like homologous neurons do in other insects (reviewed in^62^). Future work could evaluate if the function of these types of neurons are also conserved in *C*. *calcarata*.

### 4.2 Kenyon cell and astrocyte types conserved with eusocial Hymenoptera

We found two groups of Kenyon cells in *C. calcarata*: those characterized as Class A^4^ that are highly similar to the large Kenyon Cells of class I characterized in *A. mellifera* ^53^; and those similar to Class B^4^ (Figure 1). Whereas two KCB types from *D. melanogaster* ^2^ were transcriptionally similar to two *C. calcarata* subclusters from KCB, all eight *C. calcarata* KCB subclusters were transcriptionally similar to the small, medium, or the class II types of KCB identified in *A. mellifera*.^53^ The presence of KCB cell types in the *C*. *calcarata* brain suggests that Kenyon cell types are conserved beyond eusocial taxa to aculeate Hymenoptera more generally.^67^ With additional cellular neuroanatomical data from other solitary bee species, future work can test whether the *C*. *calcarata* KCB cell architecture is a shared feature among social taxa or among bees and Hymenoptera more broadly.

In addition, more subclusters of *C. calcarata* were similar to astrocyte glia cell types from social insects rather than to the single astrocyte cell type of solitary *D. melanogaster*. Social Hymenoptera brains show three astrocyte glia clusters.^1^, ^3^, ^4^ These astrocyte glia clusters show high transcriptional similarity to three different *C. calcarata* subclusters. In contrast, single‐cell brain atlases of solitary Diptera, including *Aedes aegypti and D*. *melanogaster*, show a single astrocyte cluster.^2^, ^90^ Unlike the astrocytes of eusocial Hymenoptera, the astrocyte cell type from *D. melanogaster* was highly similar to only two *C*. *calcarata* subclusters.^2^ Analyses from the brains of additional solitary and subsocial taxa are needed, however, to evaluate the extent of astrocyte cell type conservation within Hymenoptera.

### 4.3 Neuron gene expression associated with foraging

While season, reproduction and foraging affects gene expression in the whole brain of *C. calcarata*,^74^, ^91^ it was previously unclear how gene expression of *C*. *calcarata* neurons specifically responds to foraging and reproduction. The upregulation of *shab* in summer in all *C. calcarata* neurons, a gene critical to *D. melanogaster* neuron repolarization,^92^ may then reflect the elevated electrophysiological activity associated with foraging.^69^ *Apisimin*, another gene upregulated in neurons in the summer sample that also encodes a protein found in royal jelly in *A. mellifera*,^93^ shows expression across brain cells of foraging *C*. *calcarata*.^91^ In addition, we found neurons in the summer sample enriched for TORC1 signaling (Table 2), a pathway that allocates nutrients to vitellogenesis (reviewed in^94^). Neuron transcription in *C*. *calcarata* in summer then likely reflects foraging activity to support its reproduction.

### 4.4 Conservation of optic lobe gene expression associated with neurogenesis

Gene expression in the optic lobe is highly conserved among insects.^4^ Therefore, photoreceptors in *C*. *calcarata* in summer likely transduce visual cues like photoperiod to the rest of the brain (reviewed in^95^), as suggested by the expression of *D*. *melanogaster* orthologs in photoreceptors: *rdgB* relates to photoreceptor function^96^; and *slo* is needed for visual information processing.^97^, ^98^ Neurogenesis occurs in the optic lobe of adult *D*. *melanogaster*,^47^ which is also reflected in the *C*. *calcarata* optic lobe that is enriched for neuron development and various signaling pathways in summer. Neurogenesis may also be reflected by *Ctnnd2* that is upregulated in photoreceptors in *C*. *calcarata* and is a *D. melanogaster* ortholog of the gene *p120ctn* required for vertebrate nervous system development.^99^, ^100^

### 4.5 Learning associated to Kenyon cells and cognition linked to the optic lobe

In the summer sample, the optic lobe cells were enriched for cognition, whereas visual learning was enriched in Kenyon cells. Optic lobe enrichment is supported by the expression of *NaCP60E*, a sodium voltage‐gated channel responsible for information‐processing in *D*. *melanogaster*.^101^, ^102^ Activity in the optic lobe precedes selective attention to stimuli in both *A*. *mellifera* as well as *D*. *melanogaster*.^49^, ^50^ Therefore, the cognition linked to optic lobe activity in *C*. *calcarata* may be associated with the discrimination of visual stimuli. In contrast, KCA in the summer sample were enriched for neuronal function and learning, supported by expression of *slo*, a gene that facilitates calcium sensitivity in *D*. *melanogaster* neurons.^103^ Kenyon cells in summer may thus reflect the spatial learning and floral recognition of foraging *C. calcarata* mothers.^72^ Like other insects (reviewed in^104^), visual information captured by photoreceptors in *C. calcarata* is likely processed in the optic lobe before additional processing for learning in more central parts of the brain, such as Kenyon cells in the MB.

### 4.6 Reproduction reflected in astrocytes and cortex glia

*C*. *calcarata* shows elevated ovarian activity in summer,^69^ which is mirrored by the enrichment of glia for GO terms related to reproduction, such as mating and sperm competition (Table 2). In addition, two upregulated genes in summer, *Vg* and *egt*, are upregulated in the brains of reproductive adult *C*. *calcarata* as well as *A*. *mellifera* females compared with non‐reproductive ones.^64^, ^91^ *Mrjp1* is upregulated in *C*. *calcarata* glia in summer and is also involved in *A*. *mellifera* queen determination.^105^ The transcriptomic signal associated with reproduction in the *C*. *calcarata* brain therefore seems to be concentrated in glia. Within glia, the cluster of astrocyte and cortex glia cells showed enrichment for reproduction. They are also enriched for octopaminergic signaling, which is linked to reproduction in the brains of *C*. *calcarata*.^70^ Octopaminergic signaling also regulates reproduction in *D. melanogaster*,^106^ strengthening its link with reproduction in astrocytes and cortex glia of *C*. *calcarata*.

Intraspecific changes in the brain size of animals depend on many factors, like seasonality in vertebrates.^107^ Reproductive phenology is associated to shifts in neural architecture in *Ceratina* that is independent of their sociality.^108^ We extend this pattern to the cellular level, finding that *C*. *calcarata* reproductive foragers in the summer have transcriptomic signatures associated with specific brain cell types unlike inactive winter bees. Because species of solitary bees also exhibit seasonal variability in their foraging and reproduction, it is possible that they may also show transcriptomic compartmentalization to specific brain cell types at specific times of the year.

### 4.7 Winter Kenyon cells and photoreceptors linked to regulation of cell death

KCA in winter were enriched for GO terms related to cell death (Table 3). This may reflect apoptosis during overwintering as long durations of cold can cause significant cell death in *D*. *melanogaster*.^109^ Enrichment of photoreceptors in winter for the regulation of cell death may reflect cellular mechanisms to avoid cell death associated with diapause.^110^ The upregulation of *usp34* in photoreceptors in winter, a gene involved in DNA repair in *D*. *melanogaster*,^111^ may then reflect the inhibition of apoptosis after mild cold stress (reviewed in^112^).

### 4.8 Glial enrichment and gene expression in the winter sample is associated with diapause

The enrichment and gene expression in glia in the winter sample indicates diapause. All glia were enriched for both stress response and negative regulation of cell division (Table 3), which are both related to diapause. Diapause enables mitigation of cold stress,^110^ that may be reflected by the upregulation of *hsp110*, a gene involved in the freezing tolerance of the gall fly *Eurosta solidaginis*.^64^ Negative regulation of cell division may reflect the cell cycle arrest characteristic of insect diapause.^113^ This enrichment is supported by the upregulation of *Gpx4*, a gene that inhibits programmed cell death in *D*. *melanogaster*,^114^ in *C*. *calcarata* glia in winter. *Gpx4* expression in ensheathing glia is also associated with enrichment for regulation of locomotion and cell growth that reflect the decreased metabolism, apoptosis and ovarian activity associated with diapause (Table 3, ^113^). Detection of photoperiod by cells in the nervous system that express circadian clock genes can lead to insulin signaling that facilitates the expression of diapause in multiple fly species.^115^ Therefore, the enrichment of surface glia in the winter sample for GO terms related to circadian gene regulation as well as insulin signaling implicates these cells in the regulation of diapause (Table 3).

### 4.9 Limitations and future directions

The finding that cell types in the *C*. *calcarata* brain bear high similarity to those of eusocial Hymenoptera must also be considered within the context of phylogenetic distance of insects. Independent of social context, ants and bees may thus show more similar neuroanatomy to one another than to non‐hymenopteran insects.^116^ The closer transcriptional similarity of *C*. *calcarata*'s neurons and glia cell types to those of *A. mellifera*, *H. saltator* and *M. pharaonsis* than to those of the dipteran *D. melanogaster* might therefore be expected based on phylogenetic distance. Given that lifestyle as well as phylogeny influences various parts of the insect brain (reviewed in^117^), the cellular neuroanatomy of solitary bee species could help to clarify our findings.

Our measurements of transcriptional similarity relied on orthologs that can reliably distinguish conserved cell types across taxa.^80^ The use of orthologs omits taxonomically‐restricted genes that may improve the resolution of cell‐types, however. While paralogs that include taxonomically‐restricted genes have been used to estimate cell type conservation between pairs of taxa of smaller datasets,^118^ this method would not be effective for a simultaneous comparison of *C*. *calcarata* to both solitary and eusocial species.^78^ Moreover, with current bioinformatic techniques, orphan genes restricted to one species that lack homology to another species in a comparison (reviewed in^119^) cannot inform the conservation of cell‐types from single‐cell data. Therefore, future bioinformatic identification of cell types that explicitly consider these orphan genes may allow for expanded insights into the evolutionary divergence of brain cell types.

## 5 CONCLUSIONS

Using the information from a growing number of single‐cell transcriptomic insect brain atlases, we have characterized neuron, glia and hemocyte cell types in the *C*. *calcarata* brain using single‐nucleus RNA sequencing. The *C*. *calcarata* brain shares features with other insects, such as the presence of rare cell types and the proportion of neurons releasing specific neurotransmitters. The diversity of Kenyon cell subtypes and the number of astrocyte glia cell types in *C*. *calcarata* also align with those of eusocial Hymenoptera. Additional sampling from other solitary bee brains could be used to evaluate how widespread the characteristics of the eusocial brain are across Hymenoptera.

Gene expression and GO enrichment in winter *C*. *calcarata* reflects cell death and diapause in several brain cell types. When the bee was active in the summer, however, astrocytes and cortex glia showed transcription related to reproduction, while neuronal gene expression was linked to foraging. The segregation of foraging from reproduction in a subsocial brain is analogous to a social insect brain, where some cell populations express genes linked to reproduction and others show gene expression associated with foraging. The molecular neuroanatomy from solitary bees could help to clarify these findings in a phylogenetic context.

Future work in *C*. *calcarata* can examine how social experience, reproduction and foraging influences gene expression in specific brain cell types. Collectively, this work provides the first detailed single‐cell brain atlas in a subsocial insect and therefore acts as a baseline for comparison of the brains of other animals showing prolonged parental care.

## CONFLICT OF INTEREST STATEMENT

The authors declare no conflicts of interest.

## Supporting information


**Data S1:** Supporting Information.

**Figure S1.** Putative identities of *C. calcarata* cell subclusters from transcriptional similarity to known cell types in *A. mellifera*,^1^ *D. melanogaster* (^2^; Skinnider et al. 2021), *H. saltator*,^3^ and/or *M. pharaonsis* ^4^ for (A) unidentified neurons (Clusters 0,6,7 from Figure 1); (B) astrocyte and cortex glia (Cluster 5 from Figure 1); and (C) surface glia (Cluster 8 from Figure 1). Number in the legend indicates the number of the *C. calcarata* subcluster referred to as “ccalc_subcluster_num” in Table S21. AG = astrocyte glia; CG = cortex glia; OPN‐adPN = anterodorsal olfactory projection neurons; OPN‐lPN = lateral olfactory projection neurons. Indicated references correspond to those in the Supplementary methods.

**Table S1:** Supporting Information.

## Data Availability

Fastq files generated and analyzed in this study are available under BioProject PRJNA1039807 at NCBI (https://www.ncbi.nlm.nih.gov/). Code and input files used for this study are available at https://doi.org/10.5281/zenodo.12557898.
