# Supplementary material for: The brain atlas of a subsocial bee reflects that of eusocial Hymenoptera
Source: Genes Brain Behav. 2024 Nov 8;23(6):e70007. doi: 10.1111/gbb.70007 (PMC11544451; doi:10.1111/gbb.70007)
Supplement: Supplementary file 1 — Data S1: Supporting Information. [file GBB-23-e70007-s002.docx]

Supplementary Methods

*Sample collection* *and dissection*

We collected two samples of six *C. calcarata* adult female brains from Toronto, Canada. The first sample (winter; Biosample: SAMN38226699) was collected from four nests in raspberry stems on November 12, 2021 (Table S1). We chilled these nests at 4 degrees Celsius until January 24^th^-29^th^, 2022. We then collected one adult female from three different nests and three females from a fourth nest that contained three males. All six females showed minimal wing wear (median: 0) indicative of a pre-foraging status (Rehan and Richards, 2010).

We collected a second sample (summer; Biosample: SAMN38227370) on June 28, 2022 from six nests in sumac stems that we opened on the same day (Table S1). Each nest contained one reproductive mother that was caring for 3-10 larvae. These mothers also demonstrated a history of foraging with their wing wear (median: 1). In five of these nests, 1-4 eggs were also present. Head width, a proxy for the body size of female *C. calcarata* was similar between winter and summer samples (Wilcoxon test, W = 8, p = 0.103, Table S1).

One investigator (J.L.H.) extracted the brain of each bee from its head capsule. To do so, he first placed the bee on dry ice to minimize movement and nucleic acid degradation. He then removed cuticle from the head capsule with Bioquip microscissors and removed the brain from the capsule with forceps (5-SA, RubisTech, Switzerland). He then placed the brain into a sterile RNase/DNase-free 2mL cryogenic vial (BioMart, Category # 112612) that was flash frozen in a dewar of liquid nitrogen.

*Nuclei isolation and library preparation*

Personnel from the Princess Margaret Genomics Centre (PMGC) in Toronto, Canada then prepared brain tissue from each sample for single-nucleus RNA-seq analysis (snRNAseq). Brain tissue was cut into 1-2mm cubed portions using a chilled razor on dry ice and covered with lysis buffer (0.32mM sucrose, 5mM CaCl_2_, 3mM Mg(Ac)_2_, 20mM Tris-HCl 7.5, 0.1% Triton X-100, 0.1mM EDTA 8.0, 40U/mL RNase Inhibitor, and water). These tissue pieces were then cut into smaller pieces and homogenized with glass douncers for five minutes. Personnel from PMGC then stained a portion of the sample with SYBR green and evaluated nuclei quality manually with disposable hemocytometers. They then centrifuged the sample at 800g for 10 minutes and resuspended it in 2mL of wash buffer (1x PBS, 1% BSA, and 0.2 U/uL RNase Inhibitor). Then, they centrifuged the sample again, resuspended it, and centrifuged it once more. Finally, personnel filtered the sample through 40uM Flowmi cell strainer, transferred it to a 1.5mL LoBind tube placed on ice, and counted nuclei. Each sample was processed as a different batch for snRNAseq analysis.

Personnel at PMGC then loaded the sample of nuclei into 10X Genomics Gel-beads-in-emulsion wells on Chromium Chips following the protocol for Single Cell 3’ Reagent Kits (v3). After the gel beads in the sample were dissolved, nuclei were lysed, and mRNA was reverse transcribed to cDNA. Using PCR, cDNA was then amplified to create dual index paired-end libraries. Personnel checked these libraries for quality using the Agilent Bioanalyzer High Sensitivity chip. Reads 1 and 2 (R1, R2) and sample index reads (I1, I2) were then sequenced from the libraries on a flow cell lane (L001, L002, L003, or L004) on an Illumina Novaseq 6000 to create base call files.

*Read mapping*

To map the reads to the reference genome, we then input the base call files to Cell Ranger (v. 6.1.2, Zheng et al. 2017) to generate fastq files using the function mkfastq. To increase the sequencing depth (Table S2), we sequenced the same library multiple times, resulting in different fastq files for the same sample. Then, using the function count in Cell Ranger, the R1 and R2 fastq files were aligned to the most recent genome model for *C. calcarata* (Ccalcv3, BioProject: PRJNA791561) with Spliced Transcripts Alignment to a Reference (Dobin et al. 2013) to create a compressed binary representation of Sequence Alignment Map file (bam) file. Cell Ranger assigned barcodes and counted unique molecular identifiers (UMIs) for each sample. Both samples showed similar quantities of estimated cells, reads per cell, UMIs per cell, and proportions of reads mapping to the genome (Table S2). The final output of Cell Ranger included raw count matrices for genes in each cell barcode as well as matrices filtered for sequencing errors to barcodes that showed the counts of features in each cell.

*Filtering of gene matrices*

To ensure that our matrices for each sample were of high-quality, we filtered them of ambient RNA and doublets (review: Hong et al. 2022). To identify cell barcodes that were likely doublets, we ran Souporcell (v. 2.4; Heaton et al. 2020) with eight threads (t = 8) for a range of the parameter (k) corresponding to the number of clusters in each sample (k = 3-15) on the filtered barcode matrices by using the following input files: the bam file produced from STAR within Cell Ranger (possorted_genome_bam.bam); the filtered count matrix from Cell Ranger (barcodes.tsv); and Ccalcv3. The number of singlets expected from the variance in k were low for both samples (Table S3). Therefore, we used the singlets calculated at k = 15 for downstream analyses (winter: 4257 singlets; summer: 4687 singlets). Fifteen clusters would account for the following anticipated cell types identified in other studies of single-cell social insect brains: hemocytes; photoreceptors; olfactory projection neurons; astrocytes; ensheathing glia; cortex glia; three types of surface glia; four types of Kenyon cells; and non-photoreceptor optic lobe cells (Sheng et al. 2020; Traniello et al. 2020; Li, Q. et al. 2022). After importing the filtered gene expression matrix from Cell Ranger as an object into Seurat (v. 4.3.0; Butler et al. 2018) operating within R*(v. 4.2.3; R Core Team, 2023), we subset only those barcodes that were singlets identified from the souporcell analysis.

To remove counts likely to represent ambient RNA in the subset barcode matrix, we then estimated and removed counts likely to represent ambient RNA from each cell using counts rounded to the nearest integer from the decontX function in R*(package: *celda*, Yang et al. 2020). As an additional filter to remove probable empty droplets, we retained the cells in our matrix (4101 winter cells; 4394 summer cells) in R* that showed at least 500 UMIs (Seurat::nCount) and at least 200 genes (Seurat::nFeatures). These UMI and gene thresholds were used in previous studies (Sheng et al. 2020; Li, Q. et al. 2022; Zhang et al. 2022; Jones et al. 2023).

*Normalization and integration*

To account for the variance in sequencing depth among cells, we normalized gene expression counts for each cell and stabilized the variance of genes using regularized negative binomial regression with the function SCTransform (package: *sctransform*) (Hafemeister and Satija, 2019; Song et al. 2023; Church et al. 2024). After normalization, 12893 genes remained in the winter sample and 12480 genes in the summer sample.

To analyze a brain atlas common to both samples while accounting for differences in batch processing, we then integrated winter and summer samples in Seurat using established procedures (Stuart et al. 2019). We first selected the 4000 most variable genes from a list of two objects representing both samples with the function SelectIntegrationFeatures followed by PrepSCTIntegration in Seurat. Using these highly variable features, we then aligned both datasets in reduced dimensional space by performing canonical correlation analysis on both sample objects to identify pairs of cells from the two datasets that are within the same neighborhood with the function FindIntegrationAnchors. We then used the 6744 filtered anchors to integrate both samples with the function IntegrateData, producing an integrated brain atlas Seurat object of 8495 cells and 13374 genes with SCTransform-normalized counts.

*Gene annotation of* C. calcarata

We used a previous annotation of Ccalcv3 (PO1409; Brasil et al. 2023) supplemented by 82 orthologs from *A. mellifera* and 8 from *D. melanogaster* that have not been annotated in PO1409 (Table S4; Sheng et al. 2020; Traniello et al. 2020; Li, Q. et al. 2022; Zhang et al. 2022). Specifically, we identified the top hit from blastN searches according to the highest bit score (v. 2.14.0; Altschul et al. 1990; E-value threshold: 1E-6) of separate *D. melanogaster* (BioProject: PRJNA13812) and *A. mellifera* (BioProject: PRJNA471592) gene queries against a reference database of Ccalcv3.

*Clustering of integrated* C. calcarata *cell-type atlas*

To organize the nuclei of the integrated object of *C. calcarata* into clusters, we first calculated principal components of gene expression variance with the function RunPCA in Seurat. We then calculated the k-nearest neighbors for all nuclei in the integrated dataset using the first 30 principal components, based on an ElbowPlot. We then used the Louvain algorithm to estimate clusters of cells with the function FindClusters with increasing values of the resolution parameter. By executing RunUMAP, we then visualized the 30 principal components of gene expression variance across two Uniform Manifold Approximation and Projection (UMAP) axes.

*Exclusion of hemocyte cells*

One hemocyte cluster has been identified in existing cell-type atlases of the insect brain (*D. melanogaster*, Davie et al. 2018, Skinnider et al. 2021; *A. mellifera*, Zhang et al. 2022). Because clustering and subsequent cell-type identification methods use variance in gene expression (Sun et al. 2019), we wanted to exclude hemocytes to estimate cell clusters based on the gene expression variance from neurons and glia cell types in our samples. To identify hemocytes, we then measured the gene expression of two orthologs that are canonical markers of hemocytes in *A. mellifera* (*hml*, *fer2lch;* Zhang et al. 2022) across all clusters of nuclei using violin plots. With only three total clusters (FindClusters resolution = 0.006), one cluster of 145 unambiguous hemocyte cells could be distinguished. Excluding hemocytes, we then re-calculated dimension reduction of gene expression variation of the remaining cells with RunPCA to delineate neuron and glia cell types.

*Identification and labelling of glia and neuron cells*

To better distinguish neuronal and glial cell types from the dataset that had been filtered of hemocytes, we increased the resolution in the FindClusters function to find the number of clusters in which the 8350 neuron and glia cells could be unambiguously distinguished based on the relative expression of their canonical markers and visual separation, as performed in previous studies (Sheng et al. 2020; Li, Q. et al. 2022; Zhang et al. 2022). At a resolution of 0.1 that was also chosen by other studies (Sheng et al. 2020), we visibly distinguished seven neuron clusters and three glial clusters from violin plots of the normalized expression of neuron and glia markers. Using orthologs of marker genes for different cell types from the literature, we then labeled cell types corresponding to each cluster (Tables S4, S5).

To label these clusters with cell type labels, we identified genes upregulated (only.pos = T) in a cluster integrated from both samples compared to all other cells by combining p-values using the function FindConservedMarkers in Seurat (Table S5), which balances precision and recall compared to similar methods (Pullin and McCarthy, 2024). For this analysis, we specified that the gene should be upregulated in at least 10% of the cells in the focal cluster (min.pct = 0.1) because it is the minimum threshold used in similar studies (Zhang et al. 2022). We also specified a log-2-fold change in expression between the cells in the focal cluster and all other cells of at least 0.25 (logfc.threshold = 0.25), which is also used in similar studies (Li, Q. et al. 2022).

*Differential gene expression*

We performed non-parametric Wilcox Rank Sum tests to identify the genes that are differentially expressed in cell types between the winter and the summer samples with the function FindMarkers in Seurat (test = wilcox) on each cluster or groups of clusters. Because we are comparing nuclei, there are no mitochondrial or ribosomal genes in our dataset (Luecken and Theis, 2019). To evaluate the differential expression of all 13374 nuclear genes in the integrated dataset, we selected parameter values with the lowest minima for upregulation differences between samples as well as the percent of cells showing expression, as used in related work (logfc.threshold = 0, min.pct = 0; Zhang et al. 2022).

Differentially expressed genes (DEGs, Tables S6-S19) between the winter and summer samples were visualized using EnhancedVolcano plots (package: EnhancedVolcano; Blighe et al. 2019). Significant differences between the winter and summer samples in normalized expression of the 13374 genes for cells of the same cluster or groups of clusters were estimated using nonparametric Wilcox tests in Seurat. Those significantly upregulated DEGs in the summer or in the winter samples were estimated using the most inclusive criteria from previous studies for false detection rates and average change in gene expression (FDR < 0.05, Li, Q. et al. 2022; abs (avglog2fc) > 0.5, Zhang et al. 2022).

*Gene Ontology enrichment*

In preparation for Gene Ontology (GO) enrichment analysis, we then annotated the 13374 genes with GO terms using Blast2Go (v. 6.0.1; Conesa et al. 2005) as described previously (Huisken and Rehan, 2023). Specifically, each gene was queried against the nt reference using an e-value threshold of 0.001 with blastN. Then, the top hit ortholog based on e-value for each gene was mapped to a pool of GO terms by retrieving UniProt IDs from a non-redundant reference protein database. GO terms are then selected from this pool and assigned to the ortholog based on an annotation rule with the following parameters: threshold = 55; GO-weight = 5 for parent-children related terms; and an e-value-hit-filter = 1e-6.

The significantly differentially-expressed genes in each cluster or group of clusters constituted the gene lists for Gene Ontology (GO) enrichment analyses with topGO (v. 2.48.0; Alexa et al. 2006) against reference lists of genes showing counts greater than 0 in at least one cell in the integrated cluster or groups of clusters (Li, Q. et al. 2022; Sheng et al. 2020). Only the GO terms in the category of Biological Processes (BP) were used, like other studies (Sheng et al. 2020). Enrichment was calculated from a Fisher’s Exact test that compares the observed genes mapped to a specific GO term in the gene list of differentially expressed genes (‘Significant’) to those mapped to the same GO term in the reference gene list (‘Annotated’), weighted for the parent-child relationships of GO terms (algorithm: weight01). Significant GO enrichment was defined at a p-value < 0.05 (Shell and Rehan, 2022) as well as for at least 2 significant DEGs associated with the term (Li, Q. et al. 2022).

D. melanogaster *ortholog identification*

For downstream analyses, we estimated *D. melanogaster* orthologs from tblastx queries of products of all genes in datasets of *C. calcarata* (Brasil et al. 2022), *H. saltator* (Sheng et al. 2020), and *A. mellifera* (Traniello et al. 2020, 2023; Kuwabara et al. 2023) against a *D. melanogaster* reference database (BioProject: PRJNA13812). The genes of *M. pharaonsis* had already been annotated with *D. melanogaster* orthologs (Li, Q. et al. 2022). To create query databases, we used annotations and genomes from the following species: *C. calcarata* (BioProject: PRJNA791561); *A. mellifera* (BioProject: PRJNA471592); and *H. saltator* (BioProject: PRJNA445978). We found coordinates in the genome of each species using the associated annotation, and then created a fasta file with the function getfasta in bedtools (v.2.30.0; Quinlan and Hall, 2010). In the creation of the *C. calcarata* query database, we replaced the scaffold names from the genome with those used in the annotation by using the replace function in seqkit (v.2.3.0; Shen et al. 2016). For all queries, we selected the top hit of a *D. melanogaster* ortholog based on the highest bit score for each gene query and retained orthologs that showed an e-value less than 1E-5 (Li, Q. et al. 2022).

*Abundance of neurons expressing genes associated with neurotransmitter metabolism*

We used the orthologs of *D. melanogaster* to compare the proportion of neurons that serve as markers of different types of neurotransmitters among the insect brain atlases. We focused on those that indicate the synthesis and/or release of the neurotransmitters acetylcholine (*VaCHt*, *ChAT*), glutamate (*VGlut*), GABA (*VGAT*, *Gad1*), and monoamines (*Vmat*) (Table S22; Avalos et al. 2019; Allen et al. 2020; Zhang et al. 2022). The ortholog for *VaCHt* was not found in *M. pharaonsis* (Li, Q. et al. 2022). For each brain atlas, the proportion of neurons was calculated in Seurat that expressed at least one of the markers for each type of neurotransmitter.

*Subclustering procedure*

To assess the degree of analogy of *C. calcarata* brain cell types to those of other species, we compared subclusters of *C. calcarata* cell types from the integrated brain atlas to cell types that have been annotated in the brain cell-type atlases of solitary *D. melanogaster* (Davie et al. 2018) and social Hymenoptera (Table S20; *M. pharaonsis*, Li, Q. et al. 2022; *A. mellifera*, Traniello et al. 2020; Zhang et al. 2022; Traniello et al. 2023; *H. saltator*, Sheng et al. 2020). To subcluster a cell-type cluster from the integrated *C. calcarata* brain atlas, we first subset each cluster into its own Seurat object, and split each of these objects into those nuclei from the summer and winter samples (Seurat::SplitObject). To organize the nuclei in a cluster into subclusters based on the genes expressed in the cluster, we then normalized and scaled gene counts of each cluster’s Seurat object (Seurat::SCTransform) and re-calculated the 4000 most variable gene features (Seurat::SelectIntegrationFeatures). We then conducted canonical correlation analysis to identify anchors between the winter and summer samples (Seurat::FindIntegrationAnchors) and these anchors were used to integrate both samples (Seurat::IntegrateData). We used these new integrated features to calculate principal components (Seurat::RunPCA) and identify nearest neighbors with the first 30 principal components (Seurat::FindNeighbors). Using the Louvain algorithm, we then estimated subclusters of nuclei in a cluster (Seurat::FindClusters) at a resolution of 1 that has been used to sub-cluster other datasets (Avalos et al. 2019; Sheng et al. 2020) and visualized these clusters (Seurat::RunUMAP).

*Transcriptional similarity of C. calcarata subclusters to cell types from other datasets*

To distinguish more specific neuron and glia cell-types from the integrated cell-type atlas of *C. calcarata*, we then assessed the transcriptional similarity of *C. calcarata* subclusters to those of other species with Metaneighbor (v.1.16.0, Crow et al. 2018) in R*. Because marker orthologs of some cell types were not expressed in many subclusters of *C. calcarata* (Tables S27-S30), we used unsupervised Metaneighbor comparisons to evaluate the transcriptional similarity of the *C. calcarata* subclusters to cell types of at least three datasets from other species (Tables S20, S21). To avoid inflating the Type I error from multiple pairwise comparisons, *C. calcarata* subclusters were compared to all other datasets in one analysis.

To prepare for transcriptional similarity analysis, we first combined the *C. calcarata* dataset with Seurat objects of other species’ datasets that included the analyses of those studies (Table S20). After re-labelling the genes in datasets of *C. calcarata*, *A. mellifera*, and *H. saltator* with their *D. melanogaster* orthologs, shared orthologs were retained that could be identified by the intersect function of the row names of the objects in R*. Before combining individual datasets into one gene-by-sample matrix for MetaNeighbor analysis, we first converted each dataset from the format of a Seurat object into the format of a Single Cell Experiment (v. 1.20.1; Amezquita et al. 2020). We formatted one *A. mellifera* data set (Traniello et al. 2020) that was generated with earlier versions of R* packages with DietSeurat.

In order to compare the transcriptional similarity between component datasets, we then concatenated cells from each of the dataset into one larger gene-by-sample data frame, normalized the gene expression by the total expression in each cell in this data frame, grouped cells into pseudocells, and calculated highly-variable genes to create a similarity network of cells. To uniformly normalize counts from each dataset that vary in the library size for each cell in the data frame, we divided the RNA counts for a given gene in a cell by the sum of all RNA counts for all genes in a cell, and then multiplied this proportion by a factor of 1E6 (Wang et al. 2021). To account for the sparsity of the larger data frame, we then calculated pseudocells comprising the mean of cell-normalized counts of sets of 10 cells (Li, Q. et al. 2022). To identify highly-variable genes across all datasets in a pseudocell, we then used the function variableGenes (package: Metaneighbor; Crow et al. 2018). For all analyses, we found more than two-hundred genes in the larger data frame with variance in the top quartile of at least three of the component datasets. This high variability of gene expression in at least three datasets is more than 50% of the four or five component data sets being compared and is therefore appropriate (Fischer and Gillis, 2021). Using this set of highly-variable genes, we then performed unsupervised Metaneighbor analyses between subclusters of *C. calcarata* and the cell types of other species in the data frame. Area under the receiver operator characteristic curve (AUROC) scores indicated transcriptional similarity (Table S21). Specifically, AUROC values greater than 0.8 indicated significant transcriptional similarity (Li, Q. et al. 2022).

Supplement References

Alexa, A., Rahnenführer, J., Lengauer, T., 2006. Improved scoring of functional groups from gene expression data by decorrelating GO graph structure. Bioinformatics 22, 1600–1607. https://doi.org/10.1093/bioinformatics/btl140

Allen, A.M., et al., 2020. A single-cell transcriptomic atlas of the adult *Drosophila* ventral nerve cord. eLife 9, e54074. https://doi.org/10.7554/elife.54074

Altschul SF, Gish W, Miller W, Myers EW, Lipman DJ. 1990. Basic local alignment search tool. J. Mol. Biol. 215:403–410. doi: 10.1016/s0022-2836(05)80360-2.

Amezquita, R.A., et al., 2020. Orchestrating single-cell analysis with Bioconductor. Nat Methods 17, 137–145. https://doi.org/10.1038/s41592-019-0654-x

Avalos, C.B., Maier, G.L., Bruggmann, R., Sprecher, S.G., 2019. Single cell transcriptome atlas of the *Drosophila* larval brain. eLife 8, e50354. https://doi.org/10.7554/elife.50354

Blighe K, Rana S, Lewis M, 2019 EnhancedVolcano: Publication-ready volcano plots with enhanced colouring and labeling. R package version 1.2.0.

Brasil, S.N.R., Kelemen, E.P., Rehan, S.M., 2023. Historic DNA uncovers genetic effects of climate change and landscape alteration in two wild bee species. Conserv. Genet. 24, 85–98. https://doi.org/10.1007/s10592-022-01488-w

Butler, A., Hoffman, P., Smibert, P., Papalexi, E., Satija, R., 2018. Integrating single-cell transcriptomic data across different conditions, technologies, and species. Nat Biotechnol 36, 411–420. https://doi.org/10.1038/nbt.4096

Church SH, Mah JL, Dunn CW. 2024. Integrating phylogenies into single-cell RNA sequencing analysis allows comparisons across species, genes, and cells. PLOS Biol. 22:e3002633. doi: 10.1371/journal.pbio.3002633.

Conesa, A., et al., 2005. Blast2GO: a universal tool for annotation, visualization and analysis in functional genomics research. Bioinformatics 21, 3674–3676. https://doi.org/10.1093/bioinformatics/bti610

Crow, M., Paul, A., Ballouz, S., Huang, Z.J., Gillis, J., 2018. Characterizing the replicability of cell types defined by single cell RNA-sequencing data using MetaNeighbor. Nat Commun 9, 884. https://doi.org/10.1038/s41467-018-03282-0

Davie, K., et al., 2018. A single-cell transcriptome atlas of the aging *Drosophila* brain. Cell 174, 982-998.e20. https://doi.org/10.1016/j.cell.2018.05.057

Dobin, A., et al., 2013. STAR: ultrafast universal RNA-seq aligner. Bioinformatics 29, 15–21. https://doi.org/10.1093/bioinformatics/bts635

Fischer, S., Gillis, J., 2021. How many markers are needed to robustly determine a cell’s type? iScience 24, 103292. https://doi.org/10.1016/j.isci.2021.103292

Hafemeister, C., Satija, R., 2019. Normalization and variance stabilization of single-cell RNA-seq data using regularized negative binomial regression. Genome Biol. 20, 296. https://doi.org/10.1186/s13059-019-1874-1

Heaton, H., et al., 2020. Souporcell: robust clustering of single-cell RNA-seq data by genotype without reference genotypes. Nat Methods 17, 615–620. https://doi.org/10.1038/s41592-020-0820-1

Hong R et al. 2022. Comprehensive generation, visualization, and reporting of quality control metrics for single-cell RNA sequencing data. Nat Commun. 13:1688. doi: 10.1038/s41467-022-29212-9.

Huisken, J.L., Rehan, S.M., 2023. Brain gene expression of foraging behavior and social environment in *Ceratina calcarata*. Genome Biol. Evol. 15, evad117. https://doi.org/10.1093/gbe/evad117

Jones, B.M., et al., 2023. Convergent and complementary selection shaped gains and losses of eusociality in sweat bees. Nat. Ecol. Evol. 7, 557–569. https://doi.org/10.1038/s41559-023-02001-3

Kuwabara, T., Kohno, H., Hatakeyama, M., Kubo, T., 2023. Evolutionary dynamics of mushroom body Kenyon cell types in hymenopteran brains from multifunctional type to functionally specialized types. Sci. Adv. 9, eadd4201. https://doi.org/10.1126/sciadv.add4201

Li, Q., et al., 2022. A single-cell transcriptomic atlas tracking the neural basis of division of labour in an ant superorganism. Nat. Ecol. Evol. 6, 1191–1204. https://doi.org/10.1038/s41559-022-01784-1

Luecken, M.D., Theis, F.J., 2019. Current best practices in single‐cell RNA‐seq analysis: a tutorial. Mol. Syst. Biol. 15, e8746. https://doi.org/10.15252/msb.20188746

Pullin JM, McCarthy DJ. 2024. A comparison of marker gene selection methods for single-cell RNA sequencing data. Genome Biol. 25:56. doi: 10.1186/s13059-024-03183-0.

Quinlan, A.R., Hall, I.M., 2010. BEDTools: a flexible suite of utilities for comparing genomic features. Bioinformatics 26, 841–842. https://doi.org/10.1093/bioinformatics/btq033

R Core Team 2023. R: A language and environment for statistical computing. R

Foundation for Statistical Computing, Vienna, Austria (<https://www.R-project.org/>)

Rehan, S.M., Richards, M.H., 2010. Nesting biology and subsociality in *Ceratina calcarata* (Hymenoptera: Apidae). Can. Entomol. 142, 65–74. https://doi.org/10.4039/n09-056

Shell, W.A., Rehan, S.M., 2022. Comparative metagenomics reveals expanded insights into intra- and interspecific variation among wild bee microbiomes. Commun. Biol. 5, 603. https://doi.org/10.1038/s42003-022-03535-1

Shen, W., Le, S., Li, Y., Hu, F., 2016. SeqKit: A Cross-Platform and Ultrafast Toolkit for FASTA/Q File Manipulation. PLoS ONE 11, e0163962. https://doi.org/10.1371/journal.pone.0163962

Sheng, L., et al., 2020. Social reprogramming in ants induces longevity-associated glia remodeling. Sci. Adv. 6, eaba9869. https://doi.org/10.1126/sciadv.aba9869

Skinnider, M.A., Squair, J.W., Courtine, G., 2021. Enabling reproducible re-analysis of single-cell data. Genome Biol. 22, 215. https://doi.org/10.1186/s13059-021-02422-y

Song Y, Miao Z, Brazma A, Papatheodorou I. 2023. Benchmarking strategies for cross-species integration of single-cell RNA sequencing data. Nat. Commun. 14:6495. doi: 10.1038/s41467-023-41855-w.

Stuart, T., et al., 2019. Comprehensive integration of single-cell data. Cell 177, 1888-1902.e21. https://doi.org/10.1016/j.cell.2019.05.031

Sun, S., Zhu, J., Ma, Y., Zhou, X., 2019. Accuracy, robustness and scalability of dimensionality reduction methods for single-cell RNA-seq analysis. Genome Biol. 20, 269. https://doi.org/10.1186/s13059-019-1898-6

Traniello, I.M., et al., 2020. Meta-analysis of honey bee neurogenomic response links Deformed wing virus type A to precocious behavioral maturation. Sci. Rep. 10, 3101. https://doi.org/10.1038/s41598-020-59808-4

Traniello, I.M., et al., 2023. Single-cell dissection of aggression in honeybee colonies. Nat. Ecol. Evol. 7, 1232–1244. <https://doi.org/10.1038/s41559-023-02090-0>

Wang, J., et al., 2021. Tracing cell-type evolution by cross-species comparison of cell atlases. Cell Rep. 34, 108803. https://doi.org/10.1016/j.celrep.2021.108803

Yang, S., et al., 2020. Decontamination of ambient RNA in single-cell RNA-seq with DecontX. Genome Biol. 21, 57. https://doi.org/10.1186/s13059-020-1950-6

Zhang, W., et al., 2022. Single-cell transcriptomic analysis of honeybee brains identifies vitellogenin as caste differentiation-related factor. iScience 25, 104643. https://doi.org/10.1016/j.isci.2022.104643

Zheng, G.X.Y., et al., 2017. Massively parallel digital transcriptional profiling of single cells. Nat Commun 8, 14049. https://doi.org/10.1038/ncomms14049
