# Supplementary material for: The brain atlas of a subsocial bee reflects that of eusocial Hymenoptera
Source: Genes Brain Behav. 2024 Nov 8;23(6):e70007. doi: 10.1111/gbb.70007 (PMC11544451; doi:10.1111/gbb.70007)
Supplement: Supplementary file 2 — Figure S1. Putative identities of C. calcarata cell subclusters from transcriptional similarity to known cell types in A. mellifera, 1 D. melanogaster ( 2 ; Skinnider et al. 2021), H. saltator, 3 and/or M. pharaonsis 4 for (A) unidentified neurons (Clusters 0,6,7 from Figure 1); (B) astrocyte and cortex glia (Cluster 5 from Figure 1); and (C) surface glia (Cluster 8 from Figure 1). Number in the legend indicates the number of the C. calcarata subcluster referred to as “ccalc_subcluster_num” in Table S21. AG = astrocyte glia; CG = cortex glia; OPN‐adPN = anterodorsal olfactory projection neurons; OPN‐lPN = lateral olfactory projection neurons. Indicated references correspond to those in the Supplementary methods. [file GBB-23-e70007-s001.docx]

**Figure S1**. Putative identities of *C. calcarata* cell subclusters from transcriptional similarity to known cell types in *A. mellifera* (Traniello et al. 2023), *D. melanogaster* (Davie et al. 2018; Skinnider et al. 2021), *H. saltator* (Sheng et al. 2020), and/or *M. pharaonsis* (Li, Q. et al. 2022) for A) Unidentified Neurons (Clusters 0,6,7 from Figure 1); B) Astrocyte and Cortex Glia (Cluster 5 from Figure 1); and C) Surface Glia (Cluster 8 from Figure 1). Number in the legend indicates the number of the *C. calcarata* subcluster referred to as ‘ccalc_subcluster_num’ in Table S21. AG= astrocyte glia; CG= cortex glia; OPN-adPN=anterodorsal olfactory projection neurons; OPN-lPN= lateral olfactory projection neurons. Indicated references correspond to those in the Supplementary methods.
